# Structural basis of host ligand specificity change of GII porcine noroviruses from their closely related GII human noroviruses

**DOI:** 10.1080/22221751.2019.1686335

**Published:** 2019-11-12

**Authors:** Yang Yang, Ming Xia, Leyi Wang, Sahaana Arumugam, Yajing Wang, Xianjin Ou, Chenlong Wang, Xi Jiang, Ming Tan, Yutao Chen, Xuemei Li

**Affiliations:** a National Laboratory of Biomacromolecules, Institute of Biophysics, Chinese Academy of Sciences, Beijing, People’s Republic of China; b University of Chinese Academy of Sciences, Beijing, People’s Republic of China; c Division of Infectious Diseases, Cincinnati Children’s Hospital Medical Center, Cincinnati, OH, USA; d College of Life Science, Nankai University, Tianjin, People’s Republic of China; e Department of Pediatrics, University of Cincinnati College of Medicine, Cincinnati, OH, USA

**Keywords:** Emerging norovirus, viral evolution, crystal structure, viral receptor, virus-host interaction, viral attachment

## Abstract

Diverse noroviruses infect humans and animals via the recognition of host-specific glycan ligands. Genogroup II (GII) noroviruses consist of human noroviruses (huNoVs) that generally bind histo-blood group antigens (HBGAs) as host factors and three porcine norovirus (porNoV) genotypes (GII.11/18/19) that form a genetic lineage lacking HBGA-binding ability. Thus, these GII porNoVs provide an excellent model to study norovirus evolution with host ligand specificity changes. Here we solved the crystal structures of a native GII.11 porNoV P protein and a closely-related GII.3 huNoV P protein complexed with an HBGA, focusing on the HBGA-binding sites (HBSs) compared with the previously known ones to understand the structural basis of the host ligand specificity change. We found that the GII.3 huNoV binds HBGAs via a conventional GII HBS that uses an arginine instead of the conserved aromatic residue for the required Van der Waals interaction, while the GII.11 porNoV HBS loses its HBGA-binding function because of two mutations (Q355/V451). A mutant that reversed the two mutated residues back to the conventional A355/Y451 restored the HBGA-binding function of the GII.11 porNoV P protein, which validated our observations. Similar mutations are also found in GII.19 porNoVs and a GII.19 P protein mutant with double reverse mutations restored the HBS function. This is the first reconstruction of a functional HBS based on one with new host specificity back to its parental one. These data shed light on the molecular basis of structural adaptation of the GII porNoVs to the pig hosts through mutations at their HBSs.

## Introduction

Noroviruses (NoVs) are members of *Norovirus* genus in the family *Caliciviridae*. The *Norovirus* genus consists of seven genogroups (GI to GVII) that are further divided into various genotypes. Among the known NoVs, all GI, vast majority of GII, and a few strains of GIV NoVs infect humans causing epidemic acute gastroenteritis (AGE) with significant morbidity and mortality and these NoVs are referred to as human NoVs (huNoVs). The remaining NoVs infect various animal species, including bovine, swine, canine, feline, murine, ovine, Vespertilio/bat, and otarriinae/sea lion, causing gastroenteritis and/or other diseases. In addition, zoonotic infections of some animal species by huNoVs have also been observed, including rhesus monkeys [1], dogs [2], and pigs [3], through either natural or experimental infections.

NoVs recognize specific glycan ligands as host attachment factors or receptors for infection. For example, huNoVs generally bind human histo-blood group antigens (HBGAs) that play an important role in host susceptibility (reviewed in [4–6]). Similar binding phenotypes of dog and bat NoVs to HBGAs have also been reported [7,8]. Furthermore, bovine NoVs, such as Newbury 2, interact with bovine-specific α-galactoses of HBGA family [9], while murine NoVs bind ganglioside-linked sialic acids [10] as attachment receptors. However, the host ligands for the other animal NoVs, including porcine NoVs (porNoVs), remain elusive. HBGAs are complex, fucose-containing carbohydrates that distribute abundantly on the mucosal epithelia of the intestinal tract, where they most likely serve as host attachment ligands to initiate NoV infections. A recent study showed that some GII huNoVs also bound bile acids [11], a group of steroid acids that distribute abundantly in mammalian intestinal content. The bound bile acids appeared to enhance the huNoV-HBGA interactions [11], but the detailed biological significance *in vivo* remains unknown.

NoVs interact with HBGAs or other glycans through their capsid protrusions that are built by the dimeric protruding (P) domain of NoV capsid protein (VP1) [12–14]. Functional P protein dimers can be produced *in vitro* via the *E. coli* expression system [12]. Several X-ray crystallography studies showed that the recombinant P dimers are structurally indistinguishable from the authentic ones of the viral capsid [15,16]. Thus, the recombinant P dimer is a useful model to investigate NoV-glycan interactions, leading to the identification of the NoV HBGA binding sites (HBSs) and the structural basis elucidation of NoV-glycan interactions through solving the crystal structures of NoV P dimers in complex with corresponding HBGA oligosaccharides [16–24]. Furthermore, the associations between the host HBGA binding phenotypes and huNoV infection have been established through human volunteer challenge studies [25,26] and huNoV outbreak investigations [27].

**Figure 1.**
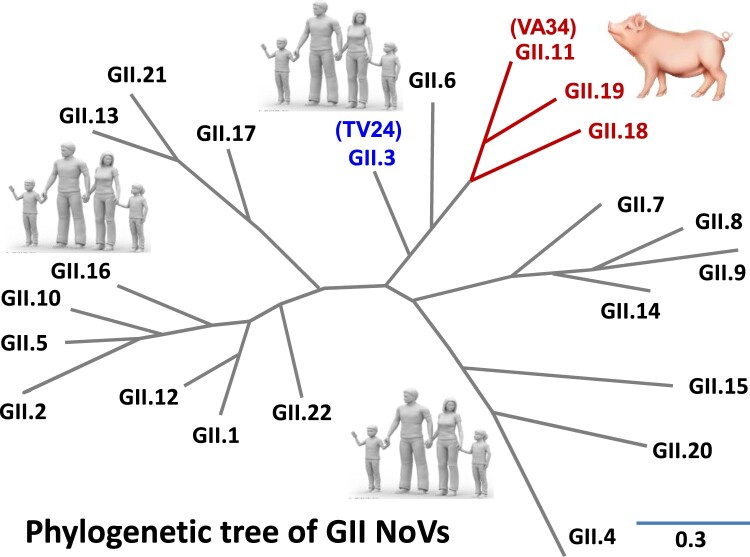
Phylogenetic tree of GII noroviruses that consist of 22 GII genotypes forming several genetic lineages. The unique lineage of GII.11/18/19 genotypes that infect pigs only is indicated by red lines and red fonts. The close-related GII.3 human norovirus is shown by blue fonts. This figure is redrawn according to the published one in [28].

Among the seven known NoV genogroups, GII is the largest one consisting of 22 genotypes (Figure 1) [28], including 19 huNoV genotypes that are responsible for the most huNoV-associated epidemics and GII.4 remains the predominant genotype in causing acute gastroenteritis in humans over the past two decades [29]. Interestingly, GII also contains three porNoV genotypes (GII.11/18/19) that form a unique GII porNoV genetic lineage, infecting most likely only pigs according to the current literature [3,30]. Phylogenic analysis indicated the GII porNoVs emerged from the GII huNoVs (Figure 1) [3,28]. The GII porNoVs were first reported in 1998 in Japan [31] and so far they have been found in domestic pig populations in numerous countries in all continents except Africa, including the USA [3], the Netherlands [32], Belgium [33], China [34,35], New Zealand [36], and Brazil [30] with various detection rates spanning from 5% to 18.9%.

PorNoV infections are generally asymptomatic with low virus shedding, although a diarrhea case of porNoV infection was reported in China [34,35]. Despite their high similarity in genomic sequences to those of GII huNoVs, particularly in VP1 sequences (∼85%) with relatively conserved HBSs [3,37,38], and the close contact between humans and pigs, there is no evidence of human infection by these G.II porNoVs. Accordingly, unlike GII huNoVs, the GII porNoVs did not bind HBGAs ([39] and the results of this report), implying that the GII porNoVs have changed their host glycan ligands from those of their GII huNoV ancestors, likely as a strategy to adapt to their new host pigs. Therefore, the GII porNoVs provide a unique model to study the molecular basis of NoV evolution with host ligand specificity changes.

In this study, we solved the P protein crystal structures of a GII.11 porNoV (VA34) that was isolated from pig caecum contents in 1997 in Japan [31] and a close-related GII.3 huNoV (TV24) that was isolated in 1977 in the USA [40], representing one of the earliest huNoV isolates, focusing on their HBSs compared with the previously known ones. We found that the GII.3 huNoV TV24 binds HBGA using a conventional HBS with an arginine instead of an aromatic amino acid for the conserved Van der Waals interaction with the HBGA fucose, while the GII.11 porNoV VA34 has two critical mutations at the HBSs, hampering its HBGA-binding function. We further showed that reverse mutations of the two mutated residues restored the HBGA-binding function of the GII.11 porNoV P protein. Similar mutations were also seen in GII.19 porNoVs and reverse mutations also helped to reconstruct functional HBS. Our data provide new insight into the molecular basis of NoV evolution with host ligand specificity change.

## Materials and methods

### Production of NoV P proteins

The cDNA fragments encoding the P proteins of the GII.3 huNoV TV24 [40] (GenBank code: U02030, amino acids 222–543 of the capsid protein), the GII.11 porNoV VA34 [31] (GenBank code: AB074893 or BAB83516, amino acid 222–542), and the GII.19 porNoV QW170 [3] (GenBank code: AAX32883.1, amino acid 222–548), respectively, were chemically synthesized and cloned into the expression vector pGEX-6P-1 and pGEX-4T-1 [Glutathione-s-transferase (GST) Gene Fusion System, GE Healthcare Life Sciences]. After transformation, the P proteins were expressed in *E. coli* BL21(DE3) strain as described elsewhere [12, 22]. The resulting GST-P protein fusion proteins were purified using glutathione-sepharose 4B (GE Healthcare Life Sciences) according to the manufacturer’s instructions and digested with Prescission (pGEX-6P-1) or thrombin (pGEX-4T-1) protease. The released P proteins were further purified by Mono Q anion ion exchange (GE Healthcare Life Sciences) at pH 8.0. Finally, the P proteins were purified by gel-filtration chromatography using column Superdex 200 (GE Healthcare Life Sciences) and the P proteins were concentrated to 10 mg/ml for crystallization.

### Crystallization of P proteins with or without oligosaccharides

The native crystals of TV24 and VA34 P proteins were grown using the hanging drop vapor diffusion method. The reservoir solution for TV24 P protein contained 9.6% (w/v) polyethylene glycol 3350, 160 mM ammonium citrate tribasic (pH 7.0), 20 mM tri-sodium citrate dehydrate (pH 5.6), 500 mM 1,6-hexanediol, and 30 mM glycl-glycl-glycin. The reservoir solution for VA34 P protein included 100 mM Tris (pH 8.0), 16% (w/v) polyethylene glycol 3350, 6% (v/v) tacsimate (pH 8.0). To obtain P protein-HBGA complex crystals, oligosaccharides representing ten different HBGAs/gangliosides at 60–100 fold molar concentration excess the P protein concentration (∼10 mg/ml) were used, including (1) A type 1 trisaccharide (GalNAcα1,3Galβ1,3GlcNAc), (2) A tetrasaccharide [GalNAcα1,3Gal(α1,2Fuc)β1,4Glc], (3) B trisaccharide (Galα1,3Fucα1,2Gal), (4) H disaccharide (Fucα1-2Gal), (5) H type 2 trisaccharide (Fucα1,2Galβ1,4GlcNAc), (6) Lewis a (Le^a^)-trisaccharide [Galβ1,3(Fucα1,4)-GlcNAc], (7) Le^b^ tetrasaccharide [Fucα1,2Galβ1,3(Fucα1,4)GlcNAc], (8) Le^x^ trisaccharide [Galβ1,4(Fucα1,3)-GlcNAc], (9) Le^y^ tetrasaccharide [Fucα1,2Galβ1,4(Fucα1,3)GlcNAc], and (10) monosialoganglioside GM1 (All oligosaccharides were purchased from Sigma-Aldrich). The TV24 or VA34 P proteins were mixed with each oligosaccharide, before co-crystallization with respective reservoir solution. Crystals can be harvested for x-ray diffraction data collection after 2 weeks at 20 °C.

### Data collection and processing

TV24/VA34 P protein native and complex (TV24 with type A tetrasaccharide) crystals were mainly mounted and collected at the BL17U beamline of the Shanghai Synchrotron Radiation Facility (SSRF) [41] and the BL5A KEK (Tsukuba, Japan) respectively. All datasets were collected under 100 K conditions and processed by HKL2000 programme [42]. The statistics of the collected data are summarized in Table 1.

**Table 1.** Statistics and refinement data of TV24 and VA34 P protein structures.

Parameters	TV24 native *P* protein (6IR5)	TV24 *P* protein complex with A tetrasaccharide (6IS5)	VA34 native *P* protein (6J0Q)
Statistics			
Space group	*P*4_2_2_1_2	*P*4_2_2_1_2	*P*4_3_2_1_2
Unit-cell parameters (Å)	*a* = *b* = 122.0	*a* = *b* = 122.4	*a* = *b* = 76.2
	*c* = 216.6	*c* = 215.2	*c* = 241.8
Wave length (Å)	1.0000	1.0000	1.0000
Resolution (Å)^a^	50.00-2.60 (2.69-2.60)	50.00-2.50 (2.59-2.50)	50.00-2.00 (2.09-2.00)
Total observation	345,842	461,448	428,954
Unique reflections	51,110	57,122	49,356
Data Completeness (%)^a^	99.9% (100.0%)	100.0% (100.0%)	99.9% (100.0%)
R_merge_ (%)^a,b^	17.1 (66.8)	16.5 (51.8)	14.0 (48.9)
I/*σ*(*I*)^a^	10.7 (2.9)	13.0 (4.5)	30.0 (7.4)
Redundancy^a^	6.8 (6.9)	8.1 (8.1)	8.7 (8.3)
Refinement			
No. of reflections in working set	50,996	57,035	46,779
No. of reflecions in test set	2,586	2,889	2,497
R_work_^c^	0.203	0.186	0.180
R_free_^d^	0.233	0.229	0.216
Root mean square deviation (*r.m.s.d*)			
Bond lengths(Å)	0.009	0.004	0.007
Bond angles (°)	0.72	0.73	1.21
Average B factors (Å^2^)			
Total	35.2	24.5	23.5
Protein	35.3	24.2	22.6
Tetrasaccharide		33.0	
Solvent	34.3	25.7	30.5
Residues in the Ramachandran plot (%)			
Favored	99.1	97.9	99.0
Allowed	0.9	2.1	1.0
Disallowed	0	0	0

^a^Values in parentheses correspond to the shell of the highest resolution.

^b^*R*_merge_ = Σi|Ii−|/ΣiIi, where Ii and are the observed and mean intensity of related reflections with common indices h,k, and l.

^c^*R*_work_=Σ||*F*_obs_|-|*F*_cal_||/Σ|*F*_obs_|, where F_obs_ and F_cal_ are observed and calculated structure factors, respectively.

^d^*R*_free_ = ΣT||*F*_obs_|-|*F*_cal_||/ΣT|*F*_obs_|, T is a randomly selected test data set (∼5%) of total reflections and was set aside before structure refinement.

### Structure determination and refinement

The Phaser programme from CCP4 [43] was used to solve the phase of the crystal structures of TV24 P protein and VA34 P protein by molecular replacement with the P protein of VA207 [22] as the searching model. The space group of TV24 P protein crystal was *P*4_2_2_1_2 and the amino acid sequence was then replaced with that of TV24 P protein. The space group of VA34 P protein crystal was *P*4_3_2_1_2 and the residues were then replaced with that of VA34 P protein. Manual model building was done using the programme COOT [44] and further refinement and adjustment were carried out with the programme Phenix [45]. Structures were validated with programme Procheck [46] and the structure analysis figures were generated by programme EdPDB [47] and PyMol [48]. The complex of TV24 P protein with type A tetrasaccharide was processed in the same way.

### Production of mutant NoV P proteins

Mutant P proteins were made as described elsewhere [14,49,50] for HBGA binding assays (below). To prove the importance of the observed HBS of the TV24 (GII.3), single amino acid mutations were introduced individually to the HBS of the TV24 P protein by site-directed mutagenesis using the expression plasmid of the wild type TV24 P protein as the template. Similarly, single and double reverse mutations were introduced to the predicted HBS of VA34 (GII.11) and GII.19 for production of mutant proteins using the expression vector of the wild type GII.11/19 P protein as the template. Mutagenesis was carried out using the QuickChange Site-Directed Mutagenesis Kit (Agilent Technology, CA) and corresponding primer pairs containing the mutation sites. Previous studies showed that a single mutation at HBS did not affect the global structure of the P protein dimer [22,38,50]. All mutant P proteins can be well detected by the hyperimmune serum against norovirus VLPs that were used in the HBGA binding assays (below).

### Glycan binding assays

Glycan binding assays were carried out as described previously [51] using oligosaccharides representing various HBGA types, well-defined human saliva samples from donors with type O, A, B, and non-secretor (N) blood types, and/or pig gastric mucin (PGM). Briefly, polyacrylamide-oligosaccharides (GlycoTech Inc) at 2 µg/mL, pretreated saliva samples (our lab stocks) at 1:1000 dilution, or type III PGM (Sigma-Aldrich) 5 µg/mL were coated on microtiter plates at 4°C overnight. After blocking with 5% (w/v) non-fat milk, the coated HBGAs were incubated with the wild type or mutant P proteins (10 µg/mL or indicated concentrations) for 60 min at 37°C. The bound P proteins were detected as described previously [21,22], using an in-house hyperimmune guinea pig serum against huNoV VLPs [51].

### Glycan arrays

Service of glycan array screenings to identify potential glycan ligands that interact with a glycan binding protein was provided by the Consortium for Functional Glycomics (CFG, http://www.functionalglycomics.org). The glycan arrays were performed using the GST-P protein fusion proteins (0.2 mg/mL) of two porNoVs, GII.11 VA34 and GII.19 QW170, respectively, against the CFG Mammalian Printed Array Library (version 5.1) that contains 609 mammalian cells associated glycans. Fluorescence labelled GST-specific antibody that was provided by CFG was used as the detection antibody. The glycan array screening results are permanently stored in the public CFG database (http://www.functionalglycomics.org/glycomics/publicdata/selectedScreens.jsp) with glycan array code: 2838.

## Results

### Glycan binding features of the TV24 and VA34 P proteins

**Figure 2.**
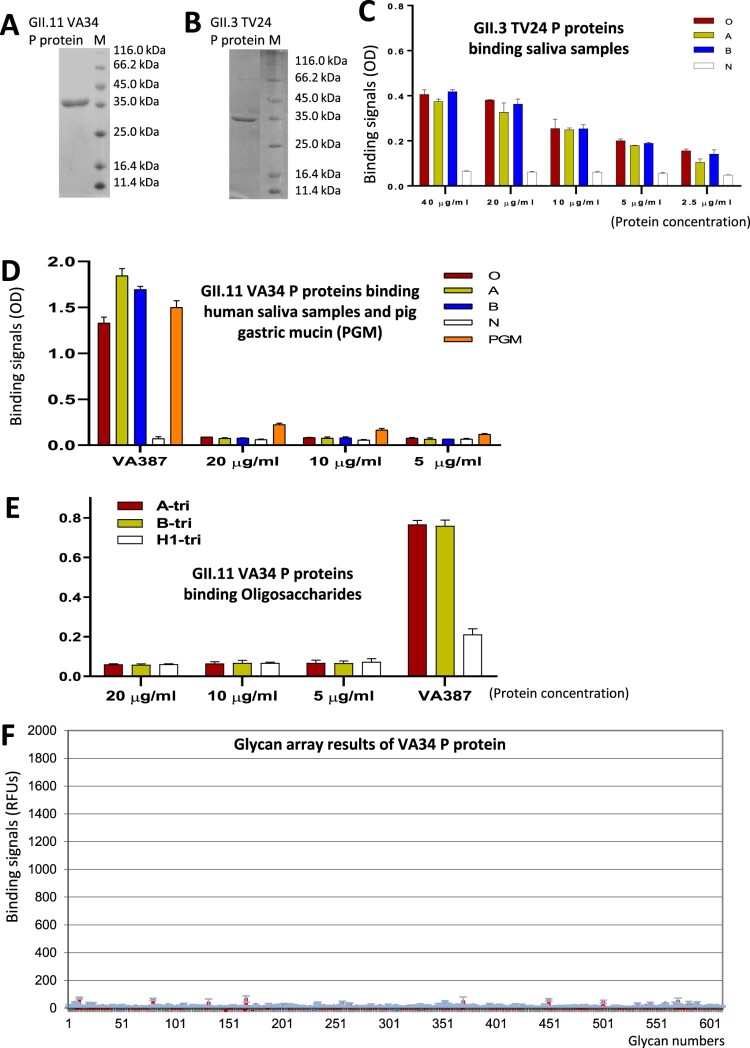
Glycan binding features of the GII.3 huNoV TV24 and the GII.11 porNoV VA34 P proteins. (A and B) Productions of the VA34 (A) and TV24 (B) P proteins via the *E. coli* expression system. The purified P proteins (∼35 kDa) are analyzed by SDS-PAGE. Lane M is the protein markers with indicated molecular sizes in kilodalton (kDa). (C to E) The binding signals in optical density (OD) at 450 nm (Y-axis) of the TV24 (C) and VA34 (D and E) P proteins at indicated concentrations (X-axis) to the four well-defined human saliva samples with type O (red), A (yellow), B (blue), and nonsecretor (N, white) HBGAs, respectively (C and D), and pig gastric mucin (PGM, orange) (D), or to three oligosaccharides representing A (red), B (yellow) and H1 (white) antigen, respectively (E). In (D and E) the GII.4 huNoV (VA387) P particles [14] at 10 µg/ml were used as positive controls. Assays in (C) and (D) were performed in the same microtiter plates thus (C) served as the positive controls for (D). (F) The results of a glycan array screening against a glycan library with 609 glycans aiming to identify glycan ligands to the VA34 P protein. The binding signals to the glycans in average relative fluorescence units (RFUs) are shown by red columns in Y-axis with standard deviations in light blue error bars, while the glycan numbers are shown in X-axis. The detailed glycan array results are stored in the public database of the Consortium for Functional Glycomics (CFG) with code 2838 (http://www.functionalglycomics.org/glycomics/publicdata/selectedScreens.jsp).

Recombinant P proteins of the GII.11 porNoV (VA34) and its closely related GII.3 huNoV (TV24) were produced (Figure 2(A and B)) to study their glycan binding features. The TV24 P protein bound secretor positive types O, A, and B human saliva samples weakly with optical density (OD) readings up to ∼0.4 (Figure 2(C)). In contrast, the porNoV VA34 P protein did not bind these saliva samples (Figure 2(D), OD < 0.1) and it did not bind the oligosaccharides representing A, B, and H antigens either (Figure 2(E), OD < 0.1). These results were consistent with the previous observations that recombinant VLPs of GII.11 VA34 did not bind any of the 52 tested human saliva samples representing different human HBGA types [39]. The ELISA-based binding assay was also performed using the porNoV VA34 P protein and commercial PGM with only low or marginal binding signals (OD between 0.1 and 0.3, Figure 2(D)). To explore other potential glycan ligands of the porNoV VA34 P protein, glycan array screenings were performed via CFG using the VA34 P protein as a probe against the CFG glycan library with 609 glycans (see Materials and methods). No specific binding signals were noted [Figure 2(F), detail glycan array results can be accessed via the CFG database (http://www.functionalglycomics.org/glycomics/publicdata/selectedScreens.jsp) with code 2838]. To show whether this is a general scenario for other GII porNoVs, we also produced the GII.19 porNoV (QW170) P protein and study its glycan binding property. Both saliva-based binding assays and glycan array screenings revealed negative results (Figure S1, detail glycan array results are stored in the same CFG database with the same code 2838) as those of the GII.11 porNoV P protein. Thus, porNoVs most likely do not bind HBGAs and thus their glycan ligands remain elusive.

### Structure of the native GII.3 TV24 P protein

**Figure 3.**
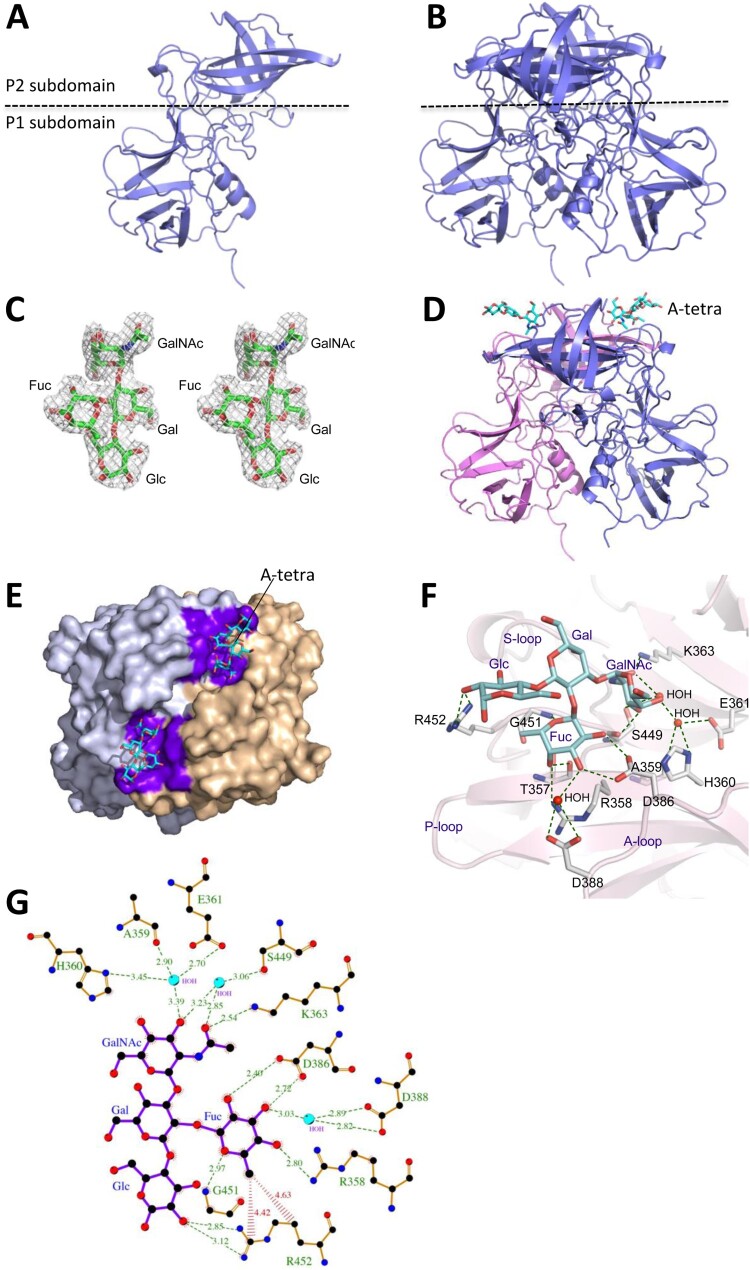
The crystal structures of the GII.3 TV24 P proteins and the HBGA binding site (HBS). (A and B) Structure of the native TV24 P protein monomer (A) and dimer (B) in the ribbon model at the side view. The dashed line shows the boundary between the P2 and the P1 subdomains. (C) Stereo view of (mFo-DFc) electron density map contoured at 2.0*σ* (grey) of the type A tetrasaccharides (A-tetra) that were calculated from diffraction intensity of complex data with the phase angle of native protein structure. (D) Side view of the P dimer-A-tetra complex. Structures of the two TV24 P proteins (ribbon model) are in magenta and blue, respectively, with indicated A-tetra (stick model) on the top. (E) Top view of the P dimer-A-tetra complex, showing the HBSs. The two P monomers (surface model) are in grey and light orange, respectively. The residues forming the HBSs are shown in purple, while the bound A-tetras (stick model) are in cyan. (F) The interaction networks between the TV24 HBS and the A-tetra. The amino acids that form the HBS are shown with labels in stick model in grey, while the A-tetra with indications of the N-acetylgalactosamine (GalNAc), fucose (Fuc), galactose (Gal), and glucose (Glc) is shown in cyan (stick model). The hydrogen bonds are shown by blue dashed lines. The A-, S- and P-loops are also indicated. (G) Ligplot of the detailed hydrogen bond (green dashed lines) and hydrophobic interactions (red lines) network between amino acids (orange) of the HBS and individual saccharides of the A-tetra (purple). Non-ligand residues located around A-tetra that do not participate in direct hydrogen bonding are shown as red shining partial circles. Water molecules are shown in cyan, carbon atoms in black, oxygen atoms in red, nitrogen atoms in blue.

The crystal structure of the native GII.3 huNoV TV24 P protein was solved to 2.6 Å resolution (Figure 3(A)), revealing P protein homodimers in the crystallographic asymmetric unit (Figure 3(B)). All P protein residues were modelled in the electron density map, except for 13 amino acids from 295 to 307 in the B-loop (see below), due to the disorder of this highly flexible long loop (Figure S2). The TV24 P dimer exhibits a typical NoV P dimer global structure.

### Structure of the GII.3 P protein-HBGA complex

The observed bindings of the TV24 P proteins to types A, B, O saliva samples prompted us to solve the crystal structure of the P proteins complexed with HBGAs. To this end, five oligosaccharides, including A type 1 trisaccharide, A tetrasaccharide (referred as A-tetra, Santa Cruz Biotechnology sc-285031), B trisaccharide (Sigma-B1422), H disaccharide (Sigma-F7012), and H type 2 trisaccharide (Sigma-F7297, see Materials and methods for their sequences) were co-crystallized with the TV24 P protein. However, only the electron density map of the A-tetra (Figure 3(C)), the only tested tetrasaccharide, was found clearly on the TV24 P protein that was solved to 2.5 Å resolution (Figure 3(D and E)). These data suggested that all four saccharides of the A-tetra may be required for a stable binding outcome, which is supported by the solved hydrogen (H) bond network between the A-tetra and the TV24 P protein (Figure 3(F and G), see below). We also tried to co-crystallize the TV24 P proteins with other oligosaccharides representing Le^a^, Le^b^, Le^x^, Le^y^ and monosialoganglioside GM1 (see Materials and methods) but none of these oligosaccharides were found to bind TV24 P protein. GM1 was tested because a previous study showed that some huNoVs bound ganglioside [52].

### The HBS of the GII.3 TV24

The crystal structure of the TV24 P dimer-A-tetra complex was solved by Molecular Replacement (MR) and showed a clear electron density map (mF_o_-DF_c_) of the extra density of A-tetra at 2*σ* contour level. All four saccharide rings are modelled clearly (Figure 3(C)), indicating the HBS at the conventional location like those of other GII NoVs [17,20,22,24], on the outmost surface of the viral capsid (Figure 3(D and E)). All four saccharides of the A-tetra interact with the TV24 P protein via 16 H bonds (Figure 3(F and G)), explaining the observation that only the tetrasaccharide formed a stable complex with the TV24 P protein. This feature also makes the TV24 HBS a large one, consisting of 16 amino acids from both P protein protomers, including those contributing to the “bottom” (T357, R358, S449 and G451) and those building the “walls” (K363, D386, D388 and R452) of the HBS.

### The Fuc-binding site

**Figure 4.**
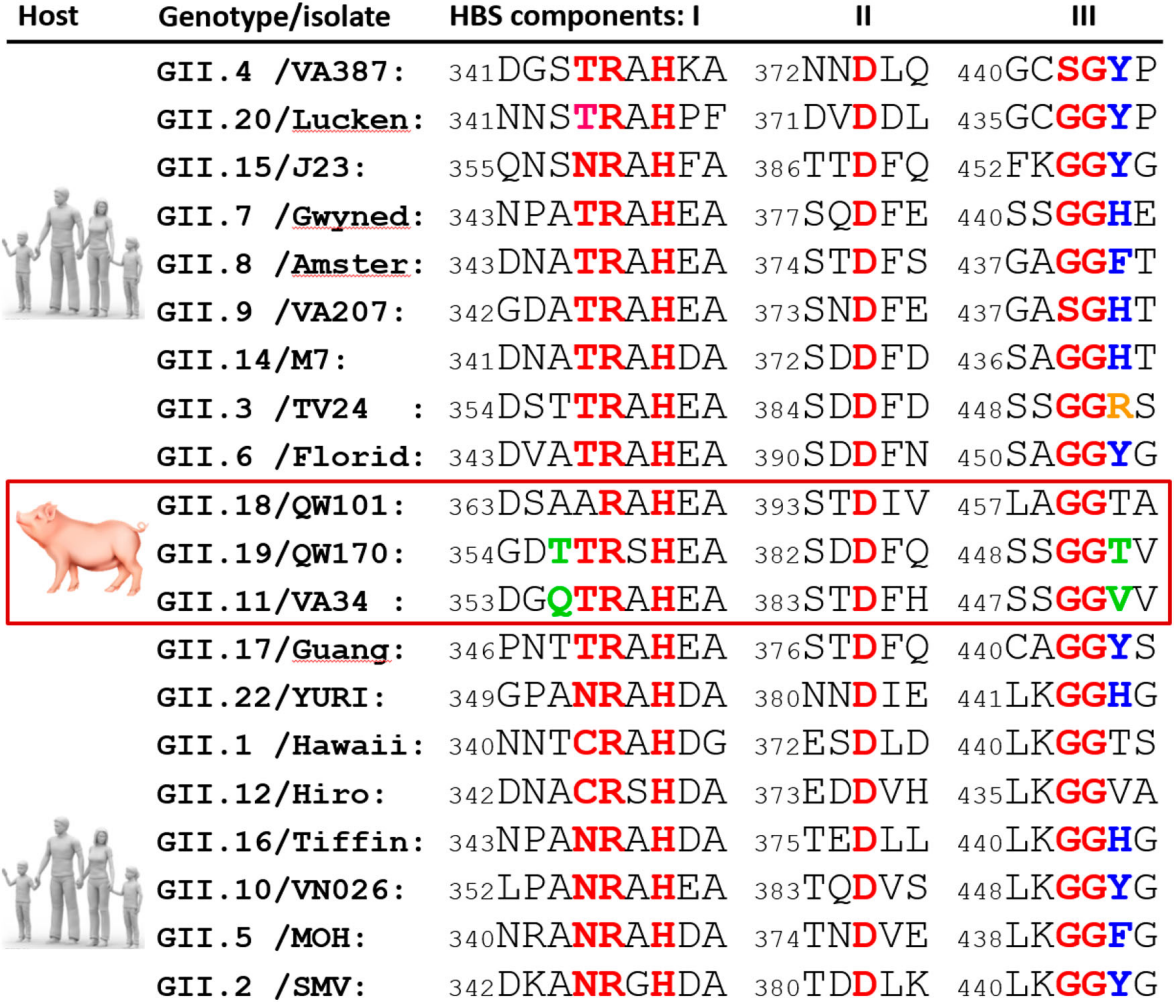
Structural-based sequence alignment among representatives of GII norovirus (NoV) genotypes, focusing on the solved (GII.4, GII.9, GII.3, GII.17, GII.12, GII.10, and GII.2) or predicted HBGA binding sites (HBSs). The sequences are arranged according to their genetic relationship shown in Figure 1, starting with the GII.4 genotype at one end and ending with GII.2 genotype at the other. The three conserved HBS components, referred as I, II and III, respectively, typically interact with the α-fucose of the HBGAs are shown in red bold letters, while their flanked sequences are shown in normal letters. The conserved aromatic residue at the end of component III that typically forms Van der Waals interaction with the α-fucose is shown in bold blue letters, while the non-aromatic arginine at this position of GII.3 (TV24), forming the same Van der Waals interaction is shown in orange. The three porcine NoV (porNoV) genotypes are framed by a red rectangle. The two residue mutations in GII.11 and GII.19 porNoVs that have been shown to damage the HBGA function are shown in green letters. For clarity, the unique genetic lineage formed by GII.13 and GII.21 genotypes that use a completely different HBS is not included in this analysis [21,53,54]. The full VP1 sequences for this analysis can be accessed from GenBank via the following codes: AY038600 (GII.4/VA387), EU373815 (GII.20/Lucken), AY130762 (GII.15/J23), AF414409 (GII.7/Gwyned), AF195848 (GII.8/Amster), AAK84676 (GII.9/VA207), AY130761 (GII.14/M7), U02030 (GII.3/TV24), AF414407 (GII.6/Florid), AY823304 (GII.18/QW101), AY823306 (GII.19/QW170), AY077644 (GII.11/VA34), KR020503 (GII.17/Guang), AB083780 (GII.22/YURI), U07611 (GII.1/Hawaii), AB044366 (GII.12/Hiro), AY502010 (GII.16/Tiffin), AF504671 (GII.10/VN026), AF397156 (GII.5/MOH), and AY134748 (GII.2/SMV).

The Fuc interacts with four highly conserved (T357, R358, D386, and G451), and two variable (T356 and D388) residues through five direct or water-bridged H bonds and some hydrophobic interactions (Figure 3(F and G)). Besides, R452 forms Van der Waals interaction with the methyl group at position 6 of the Fuc ring. While this Fuc-binding site appears to be highly conserved among most GII NoVs, we noted that, unlike R452 in TV24 HBS, most other GII huNoV HBSs show an aromatic residue (Y, H or F) at this position (Figure 4) forming Van der Waals interaction with the Fuc via their aromatic ring [4].

### The GalNAc-binding site

The GalNAc interacts with nine amino acid residues with water-involved hydrophilic and hydrophobic interactions, including A359, H360, E361, K363, S449, G450, G401, V402 and D403 (Figure 3(F and G)). A strong H bond with K363 (about 2.54 Å), and four water-bridged ones with A359, H360, E361, and S449 were found, making K363 particularly important for the A-tetra binding, but this appears not the case in other GII huNoV HBSs [20–22,24,55]. V402 and S449 form the wall of the GalNAc binding site to enhance the interactions.

### Additional interactions with Gal and Glc

Gal-Glc disaccharide is part of the HBGA precursor and these two sugars also interact with the TV24 P dimer. Particularly, Glc forms two direct H bonds to R452 and another one with a water molecule, stabilizing the TV24-A-tetra complex. This explains the only success in obtaining the stable TV24 P protein-A-tetra complex.

### Verification of the TV24 HBS

**Figure 5.**
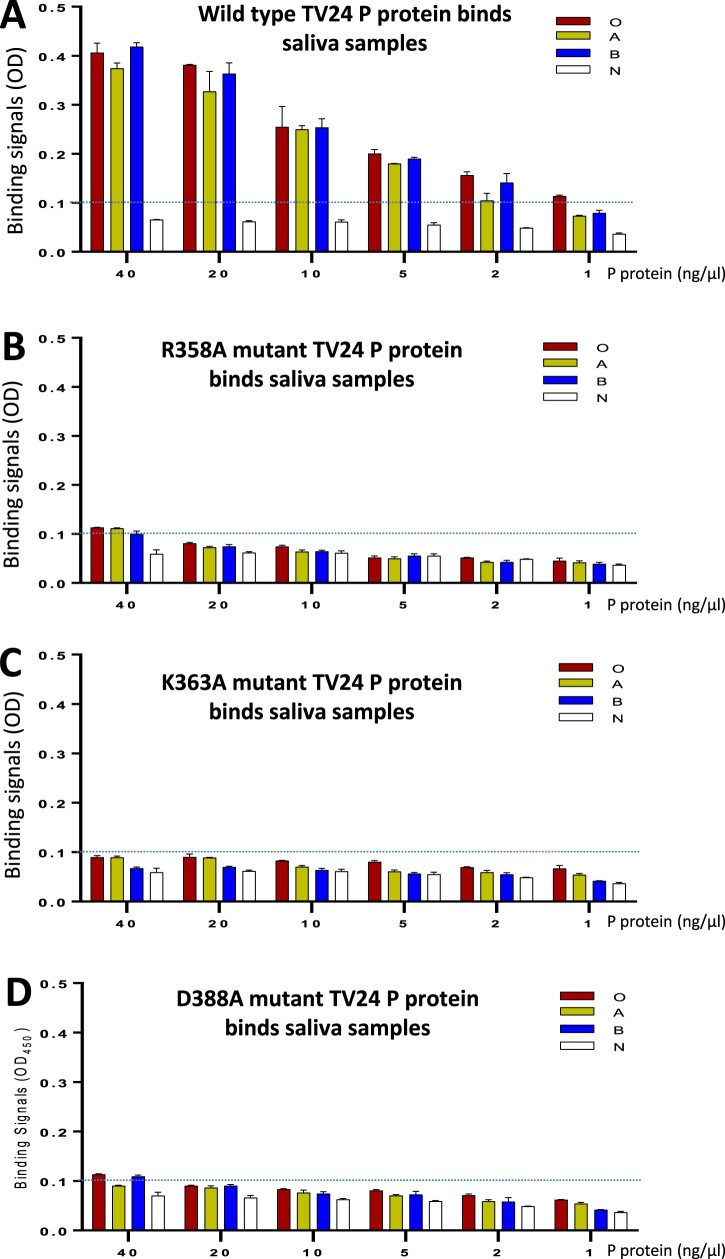
Verification of the HBGA-binding site (HBS) of GII.3 TV24 via mutagenesis. HBGA-binding abilities of the wild type (WT) (A) and three mutants’ P proteins of TV24, each having a single amino acid mutation, R358A (B), K363A (C), or D388A (D) at the HBS are determined by saliva-based HBGA-binding assays. Y-axis shows the HBGA binding signals in optical density (OD), while the X-axis shows concentrations of the P proteins. Four well defined saliva samples from donors with type O (red), A (yellow), B (blue), and non-secretor (N, white) blood types, respectively, were used. The signal cutoff (OD = 0.1) is indicated by dashed lines.

Three important amino acids (R358, K363, and D388) constituting the TV24 HBS were mutated into an alanine individually followed by evaluations of the HBGA binding functions of the mutant P proteins compared with that of wild type P protein (Figure 5). As reported previously on other GII P proteins [38,50,56], the introduction of a single mutation to the HBS did not affect the production yields and antigenic reactivities of the TV24 P protein mutants (data not shown). All three P protein mutants with a single-residue mutation at the HBS wiped out the HBGA binding capability to all type O, A, B, and non-secretor saliva samples completely or nearly completely (Figure 5), indicating the requirements of the three amino acids individually for the structural and functional integrity of the TV24 HBS.

### P protein structure of GII.11 porNoV VA34

**Figure 6.**
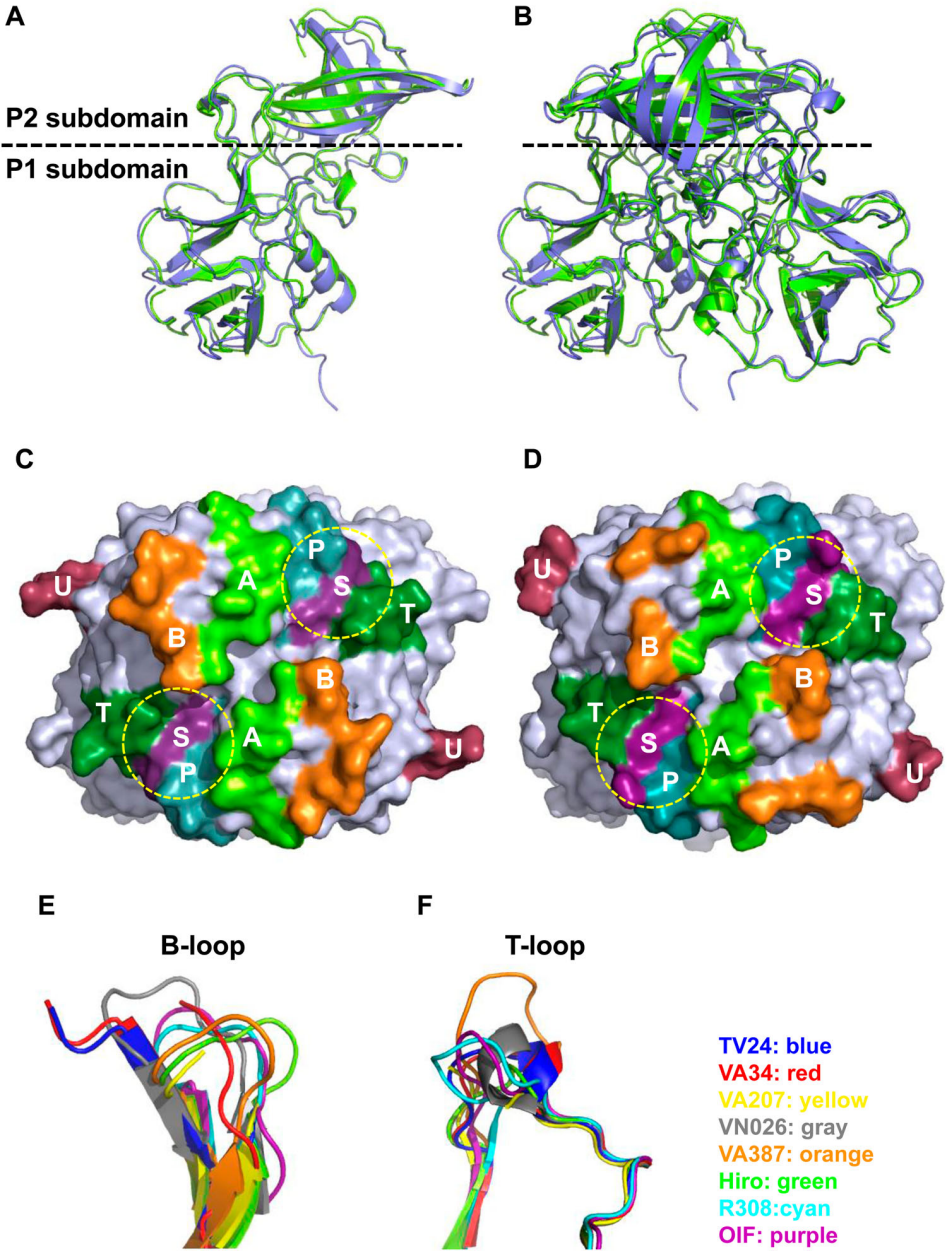
Structure of GII.11 porcine norovirus (NoV) VA34 P proteins and its comparison with that of GII.3 human NoV TV24 P proteins. (A and B) Side views of the P protein monomer (A) and dimer (B) structures of VA34 (green, ribbon model) and their superimpositions with those of TV24 P protein (blue). The dashed line shows the boundary between the P2 and the P1 subdomains. (C and D) Top views of the P dimer surface structures of VA34 (C) and TV24 (D), highlighting the six surface loops (A-, B-, P-, S-, T-, and U-loops) in different colours. The yellow dashed circles indicate the locations of the HBGA binding sites (HBSs). (E and F) VA34 and TV24 share the longest and flexible B-loop (E) and a short T-loop with a small α-helix (F) compared with the other six solved GII NoV P proteins, supporting the close genetic relationship between the VA34 and TV24 viruses.

Although VA34 P protein shows only 64% sequence identity with GII.3 TV24 P protein (Figure S2), they share similar overall structures (Figure 6(A–D)). The primary variations in both sequences and structures between the two P proteins are at the surface loops between β-strands, mainly in the P2 subdomains that form the top surface of the viral protrusions, most likely contributing to the differences in their antigenicity and HBSs. Interestingly, VA34 and TV24 P proteins share the longest B-loop compared with other GII NoVs (Figure S3), leading to the observed disorders in the crystallography and thus the unknown conformation of 13 and 12 residues of this loop (Figure 6(E)). The two P proteins also share the small T-loop with an α-helix extruding out of the flat surface (Figure 6(C–F), Figure S3). These shared structural features support the notion that the GII porNoVs are genetically closely related with the GII.3 huNoV.

### The potential glycan binding site of VA34

**Figure 7.**
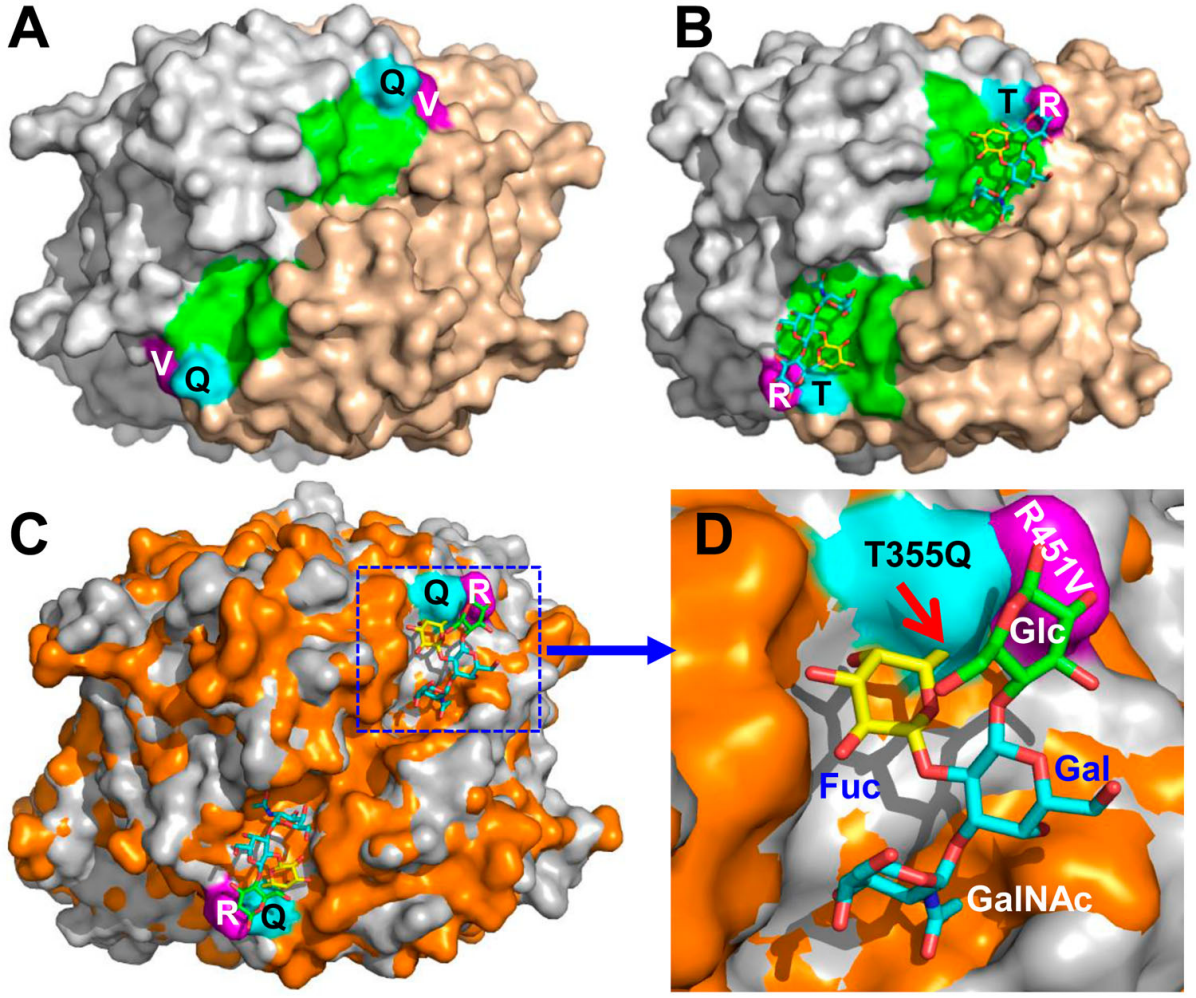
Structural comparisons of the glycan binding sites between the GII.3 TV24 and the GII.11 VA34. (A and B) Top views of the surface structures VA34 (A) and TV24 (B) P dimers (surface model with two monomers coloured as grey and sand) with the indication of the proved HBGA binding sites (HBSs, green) of TV24 (B) and the corresponding location (green) of VA34 (A). Two major mutations, T/Q355 (cyan) and R/V451 (purple) at this region of VA34 (numbered based on GII.11 VA34, see Figure 4 for details) are shown. (C and D) Superimposition of the top surface structures of the TV24 (grey) and VA34 (orange) P dimers (C) with a closeup of the HBS locations in (D), highlighting the T355Q (cyan) and the R451 V (purple) mutations (numbered based on GII.11 VA34). The type A tetrasaccharides that bind TV24 are shown in a stick model with indication of the four sugars individually. The red arrow indicates the clash between the Q355 and the Fuc.

Although 10 oligosaccharides representing various HBGAs and a ganglioside (see Materials and methods) were used to co-crystallize with the VA34 P proteins individually, no extra electron density was seen other than the native P protein, consistent with the negative results of HBGA binding assay and glycan array screening (Figure 2). However, the sequence compositions and the structure of the VA34 site corresponding to the HBSs of the TV24 and other GII huNoVs appeared to be conserved (Figures 4 and 7(A and B)), indicating that this site of VA34 is under functional selection over time during evolution. Structural superimposition of the HBS locations of TV24 and VA34 revealed two clear mutations, T355Q and R452 V, at the α-Fuc binding pocket (Figure 7(C and D)). The Q355 in the VA34 P protein leads to a steric hindrance with the methyl group of the α-Fuc, preventing the HBGA fitting into the binding pocket of the GII.11 VA34 HBS. On the other hand, the V451 mutation in the VA34 P protein loses the conserved Van der Waals interaction to the α-Fuc of the HBGAs. Thus, the two mutations damage the structure and function of the HBS and explain the lack of HBGA binding ability of GII.11 porNoV VA34.

### HBGA binding function restoration of the GII.11 VA34 P protein

**Figure 8.**
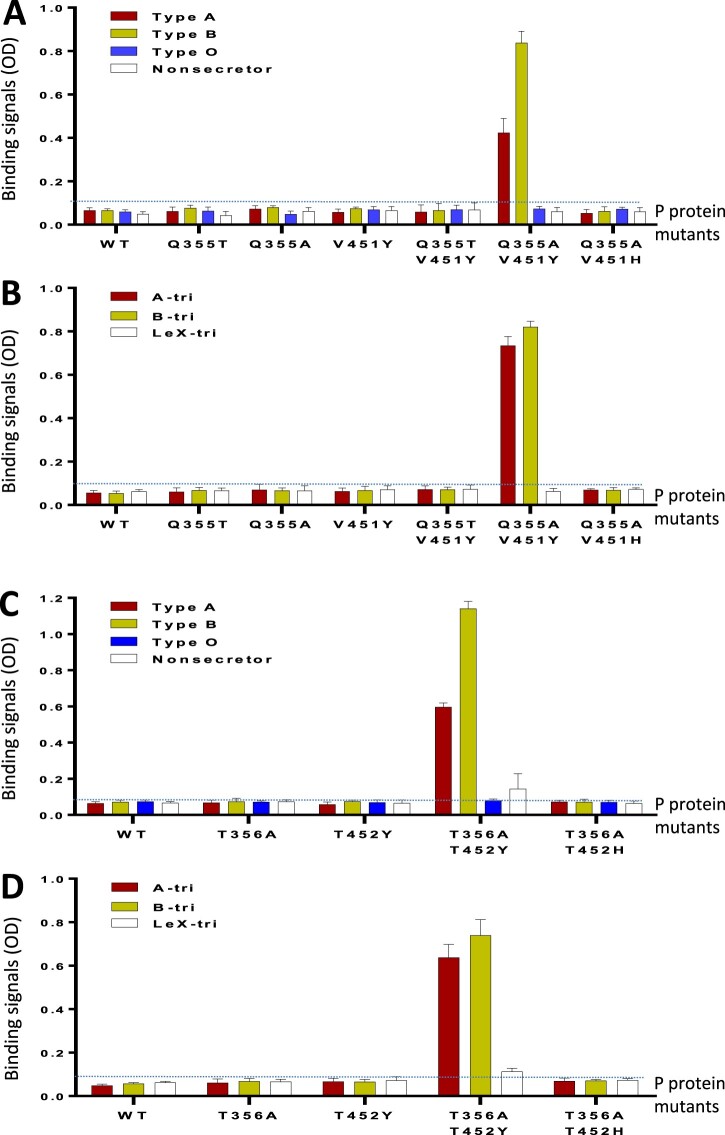
HBGA-binding function restoration of the GII porNoV P proteins via mutagenesis. (A and B) Binding of wild type (WT) GII.11 VA34 P protein and its various mutants with either single (Q355 T, Q355A, or V451Y) or double (Q355 T/V451Y, Q355A/V451Y, or Q355A/V451H) mutations at the predicted HBGA binding site (HBS) to defined types A (red), B (yellow), O (blue), and nonsecretor (white) saliva samples (A), or synthetic trisaccharide (tri) representing type A (A-tri, red), B (B-tri, yellow) and Le^x^ (Le^x^-tri, white) antigens (B). (C and D) Binding of WT GII.19 QW170 P protein and its various mutants with either a single (T356A, or T452Y) or double (T356A/T452Y or T356A/T452H) mutations at the predicted HBS to defined types A (red), B (yellow), O (blue), and nonsecretor (white) saliva samples (C), or synthetic trisaccharide (tri) representing type A (A-tri, red), B (B-tri, yellow), and Le^x^ (Le^x^-tri, white) antigens (D). The binding signals in optical densities (OD) are shown in Y-axis with error bars (standard deviations), while various WT and mutant P proteins are shown in X-axis. The signal cutoff (OD = 0.1) is indicated by dashed lines. The P protein concentration used for this study was about 20 ng/µL.

This was achieved by reverse mutations. We first made two VA34 P protein mutants, each with a single mutation to reverse the large glutamine (Q) back to a small threonine (Q355 T) or an alanine (Q355A) at this position (Figure 4) to remove the steric clash to the α-Fuc (Figure 7(C and D)). Alanine was tested because it is the highly conserved residue at this position (Figure 4). However, neither saliva- nor oligosaccharide-based binding assays showed HBGA binding ability (Figure 8(A and B)), indicating that a single reverse mutation is not sufficient to restore the HBGA binding function of the VA34 P protein, as the conserved Van der Waals interaction is still missing in these two P protein mutants with single mutations.

We then introduced an aromatic amino acid at V451 to the two VA34 P protein mutants to rebuild the missed Van der Waals interaction. The V451 was replaced with a tyrosine (V451Y) or a histidine (V451H) because these two aromatic amino acids occur most frequently at this position (Figure 4). This led to three VA34 P protein mutants each with double mutations: (1) Q355 T/V451Y, (2) Q355A/V451Y, and (3) Q355A/V451H. Both saliva- and oligosaccharide-based binding assays showed that the Q355A/V451Y mutant gained binding function to A and B antigens (Figure 8(A and B)), indicating that, only specific double reverse mutation (Q355A/V451Y) can restore the HBGA binding function of the VA34 P protein. Finally, we also made a VA34 P protein mutant with a single V451Y mutation. This mutant did not show HBGA binding function, indicating that rebuilding the Van der Waals interaction alone did not restore the HBGA binding capability of VA34 P protein.

### HBGA binding function restoration of the GII.19 P protein

To explore whether the above HBS restoration principle applies to other porNoVs, we made four P protein mutants of GII.19 QW170 based HBS sequence alignment (Figure 4). Two had a single mutation each (T356A or T452Y), while the other two had double mutations each (T356A/T452Y or T356A/T452H) (Figure 8(C and D)). Both saliva- and oligosaccharide-based HBGA binding assays showed that the T356A/T452Y P protein mutant gained HBGA binding function to A and B antigens, while the T356A/T452H P protein mutant did not (Figure 8(C and D)), a scenario similar to that of the GII.11 VA34.

## Discussion

By solving and comparing the crystal structures of a GII.11 porNoV P protein and a closely related GII.3 huNoV P protein in complex with an A-tetra, we illustrated the molecular basis of the host ligand specificity changes from HBGAs for GII.3 huNoVs to an as-yet-unknown factor for GII.11 porNoVs. Although the GII.3 huNoV TV24 binds HBGAs using the conventional GII HBSs, unlike many other GII huNoV HBSs, the GII.3 TV24 P protein interacts with all four terminal saccharides of the A antigen and both the α-Fuc and GalNAc appear to play equally important roles in the binding outcome. In addition, the GII.3 TV24 HBS is unique as it has an arginine (R452) at the HBS component III, unlike the aromatic residue in other GII HBSs (Figure 4), but still forms the conserved Van der Waals interaction with the α-Fuc of an HBGA. We found that the GII porNoVs lose their HBGA binding ability via two critical mutations at their HBSs and have succeeded to restore the HBGA binding function in both GII.11 VA34 and GII.19 QW170 P proteins through specific double reverse mutations of the two mutated amino acids, validating our observations and providing insight into the NoV evolution with host specificity change.

A basic question is whether the GII porNoV HBSs remain functional and if yes, what they may bind. In our study, we showed that the GII porNoV P proteins did not bind HBGAs, as shown by saliva- and oligosaccharide-based HBGA binding assays, which was further supported by co-crystallization, followed by X-ray crystallography. We noted weak or marginal binding signals of the GII porNoV to PGM, suggesting that the GII porNoV HBSs could remain functional at a low level. However, the glycan array screenings, aiming to identify specific glycan ligands that interact with the GII.11 (VA34) or GII.19 (QW170) P proteins were not able to identify any specific glycan ligand. The GII.11 VA34 P protein did not reveal clear binding signals, while GII.19 QW170 P protein only showed some weak and inconclusive signals. Thus, the ligands for the GII porNoVs remain elusive.

Despite these negative or inconclusive results, the following two observations also support the notion that the GII porNoV glycan binding sites may be functional. First, the amino acids constituting the GII porNoV glycan binding sites remain highly conserved (Figure 4), indicating that they are under strong functional selection pressure over time. A glycan binding site without such functional selection would lose the conservations. For example, the site of the conventional GII HBS in the GII.13 and 21 genotypes loses its conservation, as a result of obtaining a completely new HBS [21]. Second, porNoVs remain infective and circulate in domestic pig populations all over the world and they must be able to recognize certain host attachment factors for infection as other NoVs do. Based on the observed weak binding signals of the GII porNoV to PGM, we speculated that such host factors should be pig-specific because the porNoVs have never been reported to infect humans or other animal species. We also speculated that the attachment factors are glycans because the glycan binding sites of porNoVs remain conserved (Figure 4) and likely functional (see above). Thus, the porNoVs must have adapted to a new, pig-specific glycan via the two observed amino acid changes at the glycan binding site. Future study is necessary to identify the host ligands of porNoVs. Since GII porNoVs are mostly non-pathogenic and they were mostly found through a systematical screening of farmed animals, future study should include systematical screenings of human stool samples for GII porNoVs to examine whether porNoVs can infect humans.

The success in restoration of the HBS function of the porNoV P proteins to both A and B antigens verified the notion that the two mutated residues damaged the HBS function. This finding, in combination with the previously developed plasmid-based reverse genetics system for huNoVs [57], may provide a feasible platform to generate porNoV mutants with HBGA binding phenotypes using the known full genome sequences of porNoVs [34,58] and mutagenesis to study the roles of host factors and host specificity change of porNoVs. Since domestic pigs are known to be A-antigen positive, it would be of significance to test whether the porNoV mutant with A-antigen binding capability would still infect or even increase the infectivity of the mutant porNoVs compared with the wild type ones. Due to a safety concern that such mutant porNoVs could gain an ability to infect humans, an alternative approach would be to modify the HBSs of huNoVs to the form of porNoV HBSs, followed by an examination of possible improved infectivity of the mutant huNoVs in pigs. The outcome would shed light on the roles of host ligands in the infectivity and host specificity of NoVs.

Genetic analysis based on the genome sequences indicated that the GII porNoVs emerged from a GII huNoV ancestor as they share the highest sequence homology [3,28] (Figure 1). However, how the porNoV occurred remains a puzzle. The fact that the porNoVs lose their HBGA binding ability and that they infect pigs only suggested that the porNoVs have changed their host attachment factors and host specificity. The structural basis for the loss of the HBGA binding ability has been clearly illustrated, which is due to the mutations of the two specific amino acids at the glycan binding site, resulting in 355Q/451 V in GII.11 VA34 and 356 T/452 T in GII.19 QW170. This should provide the structural basis for the binding function to the new host attachment factors, most likely pig-specific glycans (also see above).

The failure of our glycan array screening to identify the glycan ligands of porNoV P proteins may be due to two reasons. First, the porNoV P protein may bind a glycan weakly, as shown by the marginal signals in both the HBGA binding arrays and the glycan array screenings. If this is true, it could explain the fact that porNoV infections do not generally cause a symptom and the virus load in the stool of infected pig is generally low, indicating a low replication rate after infection. Alternatively, the negative results of glycan array screenings may also mean that the specific glycan ligands of the porNoVs are not included in the 609 glycans of the CFG glycan library. Thus, the glycan ligand(s) of the porNoVs need to be defined by future studies.

The authors of a recent study claimed that they solved the crystal structure of a GII.19 huNoV P protein in complex with a bile acid [11]. This is confused as GII.19 should not be a huNoV but a porNoV genotype and we noted that both sequences and structure of the “GII.19 huNoV” P protein differ greatly from our GII.11 porNoV P protein. These prompted us to perform further analyses to clarify this confusion. Genotyping the “GII.19” sequences (AB083780) used in that study [11] via the online Norovirus Typing Tool at NoroNet (https://www.rivm.nl/mpf/typingtool/norovirus/) showed that the NoV with the code of AB083780 is a GII.P22/GII.22 huNoV, not a GII.19 porNoV. Accordingly, BLAST search via NCBI (https://blast.ncbi.nlm.nih.gov/) also showed that the AB083780 sequence clustered with those of GII.P22/GII.22 huNoVs. Thus, our crystal structure of the GII.11 VA34 P protein represents the first solved P protein structure of a GII porNoV.

Some unique features of the GII.3 TV24 HBS were noted compared with other GII huNoV HBSs. Unlike most known huNoV HBSs, TV24 HBS interacts with all four terminal saccharides of the A-antigen mainly via the H bonds, among which both the α-Fuc and the GalNAc contribute significant interactions to the binding outcome. This explains why electron density maps of oligosaccharides are only from the A-tetra, as the Glc may also be required for a stable binding outcome of the tested oligosaccharides. The failure to obtain an electron density map for the H-trisaccharide that contains the Glc remains unclear, probably due to the lack of the GalNAc that exhibits strong interactions with the HBS. Alternatively, other saccharide(s) of the H antigen in saliva sample may also contribute to the observed binding results of a saliva-based binding assay. The native HBGAs generally contain more sugars than those in the synthetic oligosaccharides.

Another unique feature of the TV24 HBS is the arginine at position 452 (R452) that forms Van der Waals interaction with the α-Fuc of an HBGA. The previously determined structures of the GII HBSs and the sequence alignment (Figure 4) of GII HBSs showed that the conserved Van der Waals interaction at this position generally forms between the α-Fuc and the aromatic side chain of amino acid, mostly a tyrosine or a histidine. The positively charged arginine at this position is the only non-aromatic residue that has been shown to form the conserved Van der Waals interaction with the α-Fuc, which appears to be required to support a stable HBGA binding outcome. Consistent with this notion, GII.1 NoV such as Hawaii virus that lacks this aromatic residue (Figure 4) does not bind HBGA [51]. Similarly, the GII.12 that lacks this aromatic residue was shown to bind HBGA weakly [24]. Finally, the new GII.17 variant with tyrosine at this position binds HBGAs strongly, while the previously circulated GII.17 variant lacking such an aromatic residue does not bind or bind weakly to HBGAs [53].

Although the GII HBSs generally need a small amino acid (A, T, or S) at the position 355 and an aromatic residue (Y, H, or F) at the position 451 (numbered based on GII.11 VA34) (Figure 4), only specific amino acids at these two positions make the HBS functional. Via the mutagenesis study, we found that the alanine with a hydrophobic side chain helped to rebuild the porNoV HBS, while a threonine with polar side chain did not (Figure 8). Similarly, a tyrosine at position 451 helped to restore the porNoV HBS function, while a histidine did not. These data explain the scenario why we see mostly an alanine but occasionally also a threonine or a serine in some cases at the position 355. Likewise, our results explain why the GII HBS need a tyrosine mostly, but it could also be a histidine or phenylalanine, or even an arginine (Figure 4), depending on the detail combinational need of a given GII HBS.

## Supplementary Material

Supplemental Material
