# Supplementary figures and images for: Structural basis of host ligand specificity change of GII porcine noroviruses from their closely related GII human noroviruses
Source: Emerg Microbes Infect. 2019 Nov 12;8(1):1642–57. doi: 10.1080/22221751.2019.1686335 (PMC6853222; doi:10.1080/22221751.2019.1686335)

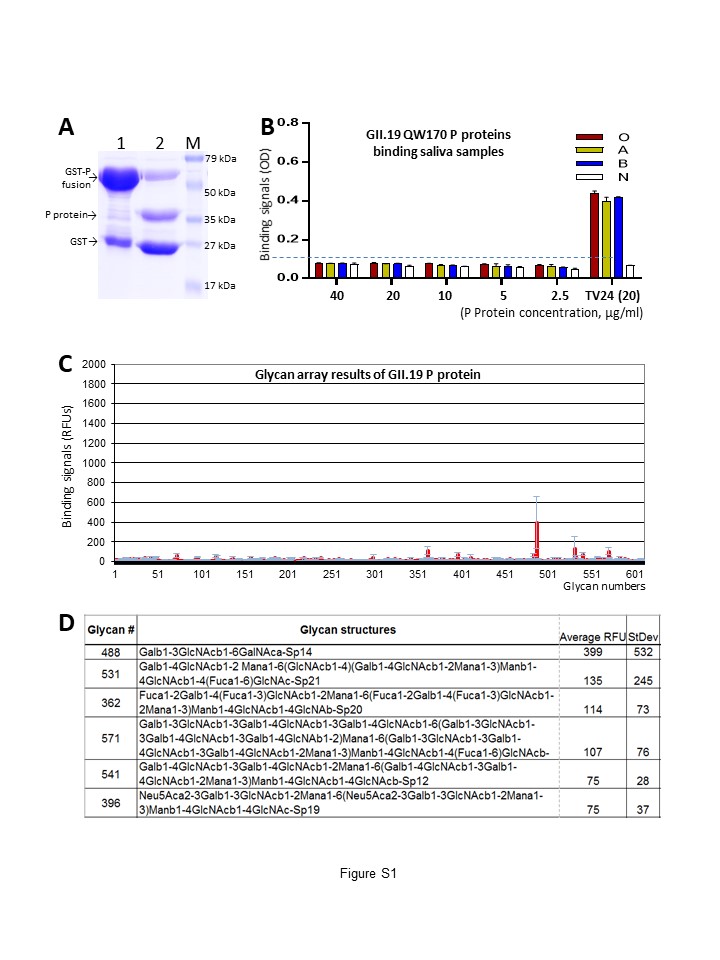

Supplement: Supplemental Material [file TEMI_A_1686335_SM5378.zip › Figure_S1_final.JPG]

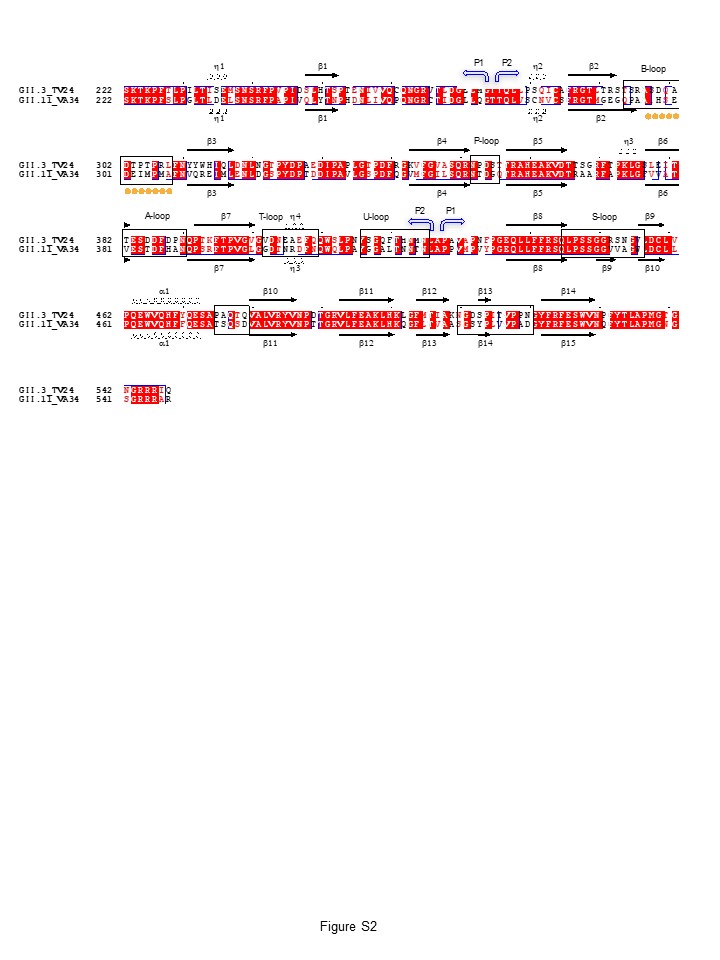

Supplement: Supplemental Material [file TEMI_A_1686335_SM5378.zip › Figure_S2_final.JPG]

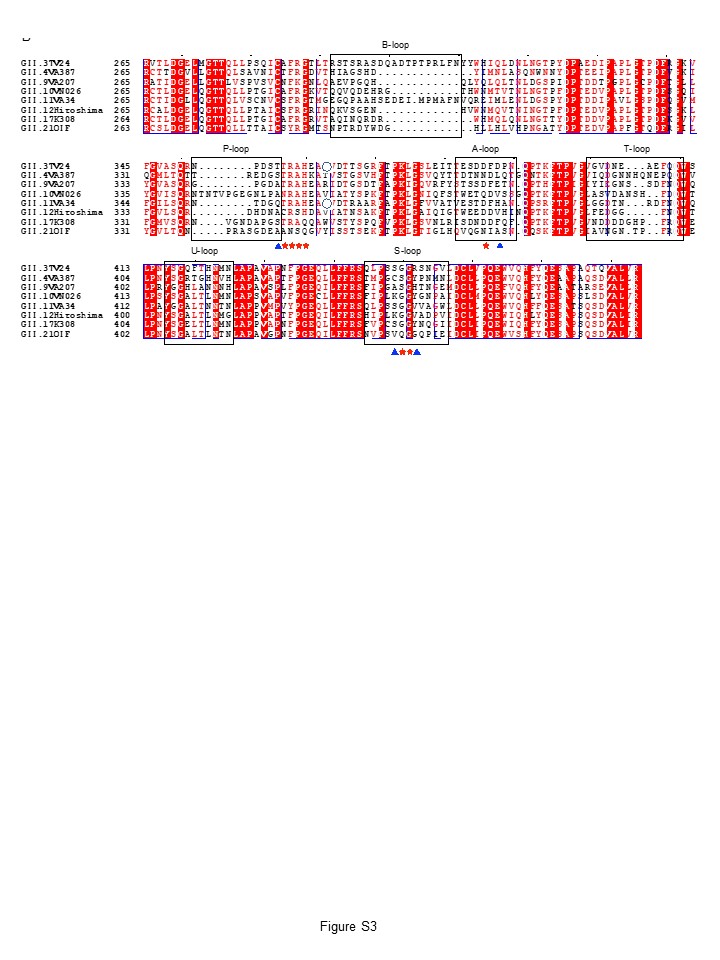

Supplement: Supplemental Material [file TEMI_A_1686335_SM5378.zip › Figure_S3_final.JPG]
